# A Rare Case of Hao-Fountain Syndrome Mimicking Fragile X Syndrome

**DOI:** 10.7759/cureus.45332

**Published:** 2023-09-15

**Authors:** Khaled N Itani, Salma Elfaki

**Affiliations:** 1 Osteopathic Medicine, Lake Erie College of Osteopathic Medicine, Bradenton, USA; 2 Pediatrics, Nona Pediatric Center, Orlando, USA

**Keywords:** fragile x syndrome, inattentive adhd, lisdexamfetamine, intellectual disability, café au lait spot, dysmorphic facies, adhd, whole exome sequencing, usp7, hao-fountain syndrome

## Abstract

Hao-Fountain syndrome (HAFOUS) is a rare neurodevelopmental disorder caused by mutations in the ubiquitin-specific protease 7 (USP7) gene for endosomal recycling. The diagnosis is often challenging due to the nonspecific presentation of intellectual disability and developmental delay, often accompanied by dysmorphic facies. In this case, we present an 18-year-old female with intellectual disability (ID), attention-deficit/hyperactivity disorder (ADHD), and dysmorphic facies who had undergone single nucleotide polymorphism (SNP) microarray and fragile X polymerase chain reaction (PCR) testing five years prior to diagnosis, both returning with negative results for genetic anomalies. The patient was managed symptomatically for ADHD until recently when the topic of a possible genetic condition was reintroduced to the family, who were agreeable to a referral to a medical geneticist and repeat genetic testing. Repeat testing, but now with whole-exome sequence (WES) analysis, revealed a pathogenic variant of the USP7 gene, prompting the diagnosis of Hao-Fountain syndrome. Our patient continues to be symptomatically managed for ADHD and intellectual disability. Educational resources and support group information were also shared and discussed with the patient and her family in the wake of this rare diagnosis.

## Introduction

Hao-Fountain syndrome (HAFOUS) is a rare neurodevelopmental syndrome caused by mutations in the USP7 gene for endosomal recycling. It was first described by Hao et al. in 2015 [1]. According to the Foundation for USP7-Related Diseases, approximately 200 cases of this syndrome have been reported globally; there are currently no standardized management or treatment guidelines. The USP7 gene located on chromosome 16p13.2 codes for a deubiquitinating enzyme currently believed to be involved in androgen receptor responsivity in certain cancers in males, embryonic implantation in females, genitourinary development, cell cycle regulation, and stabilization of tumor suppressors [1-3]. In a 2022 paper, Priolo et al. compiled the most common clinical features of HAFOUS, with the most common signs and symptoms observed being speech delay, developmental delay/intellectual disability (ID), and dysmorphic facies [2]. Specifically regarding facial dysmorphia, the most common patterns are deep-set eyes, long palpebral fissures, and a low-hanging or prominent nasal septum [2,4]. Additionally, in their study of 17 patients examined, nine and seven individuals displayed attention-deficit/hyperactivity disorder (ADHD) and autism spectrum disorder (ASD), respectively [2]. As with many Mendelian genetic disorders, whole-exome sequencing (WES) forms the basis for establishing a definitive diagnosis [3,4].

In this case report, we present an 18-year-old Hispanic female with mild ID, ADHD, and facial dysmorphia who underwent genetic testing for fragile X syndrome (FXS) along with a single nucleotide polymorphism (SNP) microarray in 2018, which revealed no anomalies. However, in 2023, on repeat genetic testing but now utilizing WES, she was found to have a pathogenic variant of the USP7 gene, leading to the diagnosis of HAFOUS.

## Case presentation

The patient is an 18-year-old Hispanic female who transferred to this pediatrics practice in 2021 after moving from another city. Parents provided documentation from a neuropsychology practice in 2018 showing results for a series of cognitive assessments where she performed below the ninth percentile on most, with the majority being 1st percentile or below, along with an intelligence quotient (IQ) of 59 (first percentile). She was diagnosed with ID and inattentive ADHD and recommended for an individualized education plan (IEP), speech-language and occupational therapy, and psychotropic medication initiation. At the time, the patient was prescribed dexmethylphenidate (Focalin®); however, she did not experience improvement in ADHD symptoms. She was later switched to lisdexamfetamine (Vyvanse®) 20mg. The patient had also undergone genetic testing with SNP microarray (Reveal®) and fragile X polymerase chain reaction (PCR) with reflex Southern Blot, both of which were negative for genetic anomalies.

The patient presented to this practice with no active medications at the time and parental complaints of hyperactivity, impulsivity, inattention, and difficulty in school consistent with her diagnoses of ADHD and ID. On the physical exam, the patient was also noted to have low-set ears, no nasal anomalies, deep-set eyes, as well as small hands and feet. The patient lived with her mother and stepfather; the biological father was not involved in the patient’s life and his medical history was unknown. The patient’s mother has a healthy adult daughter from another relationship and denied any medical history in her family; consanguinity with the biological father was denied. The patient’s mother reported that the patient had early developmental delays, citing that the patient never crawled and did not start walking until the age of two; speech delays, in which the patient did not start using single words until the age of four and did not start speaking phrases until the age of six; as a child, the patient received speech therapy. The patient's mother also reported that the patient had difficulty feeding as a child and ate only blended foods until the age of four due to an aversion to chewing as well as chronic constipation. At the time the patient had no longer been receiving treatment for ADHD, it was decided the patient would undergo pharmacogenomic testing (Genomind®) to help determine a proper regimen efficiently (Table 1).

**Table 1:** Dopaminergic stimulant excerpt from the pharmacogenomic report COMT: catechol-O-methyltransferase; ADRA2A: adrenoceptor alpha 2A

Medication	Pharmacodynamic association	Pharmacodynamic gene	Pharmacokinetic gene
Amphetamine-dextroamphetamine	Higher odds of response	COMT	2D6
Dexmethylphenidate	Lower odds of response	ADRA2A	-
Dextroamphetamine	Higher odds of response	COMT	2D6
Lisdexamfetamine	Higher odds of response	COMT	2D6
Methamphetamine	Higher odds of response	COMT	2D6
Methylphenidate	Lower odds of response	ADRA2A	-

**Figure 1:**
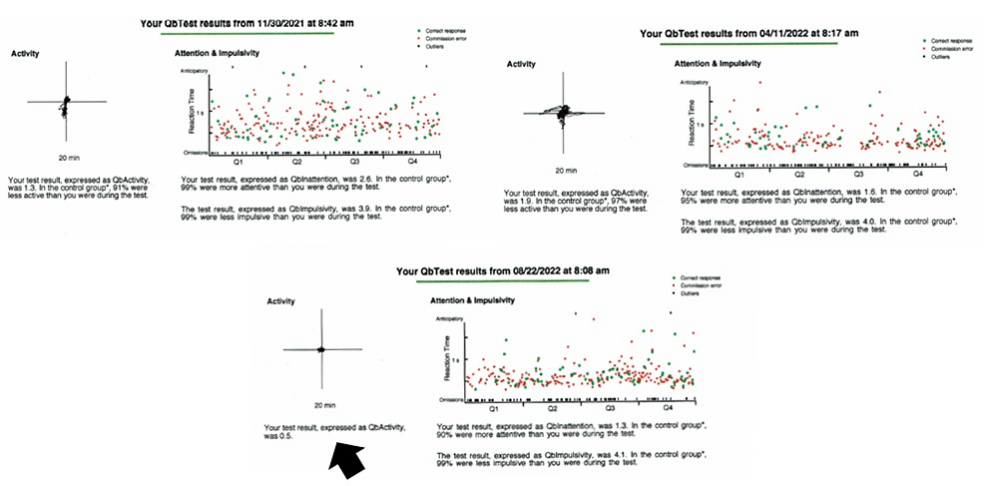
QbTest® demonstrates dose optimization from lisdexamfetamine 10mg to 30mg. Dose optimization for hyperactivity August 22, 2022

Interestingly, in her pharmacogenetic report, she displayed pharmacodynamic and pharmacokinetic genes yielding lower odds of response for dexmethylphenidate (Focalin®), which corresponded to her clinical response years prior to testing. It was decided to restart the patient on lisdexamfetamine (Vyvanse®). Over the following months, measures for dose optimization were gathered via periodic QbTest® and symptomatic descriptions from the patient and her family. In August 2022, it was determined that dose optimization for hyperactivity had been reached with lisdexamfetamine (Vyvanse®) from 10mg to 30mg; less pronounced variable improvement in the domains of inattention and impulsivity was attributed to underlying unknown genetic causes of neurocognitive pathology (Figure 1).

Five years ago, the patient underwent SNP microarray and fragile X PCR testing, both of which did not reveal genetic anomalies. The patient and her family agreed to a referral to a neuropsychologist and medical geneticist in order to further investigate the etiology of her treatment-refractory inattention and impulsivity, as well as her previously unexplained facial dysmorphia.

**Figure 2:**
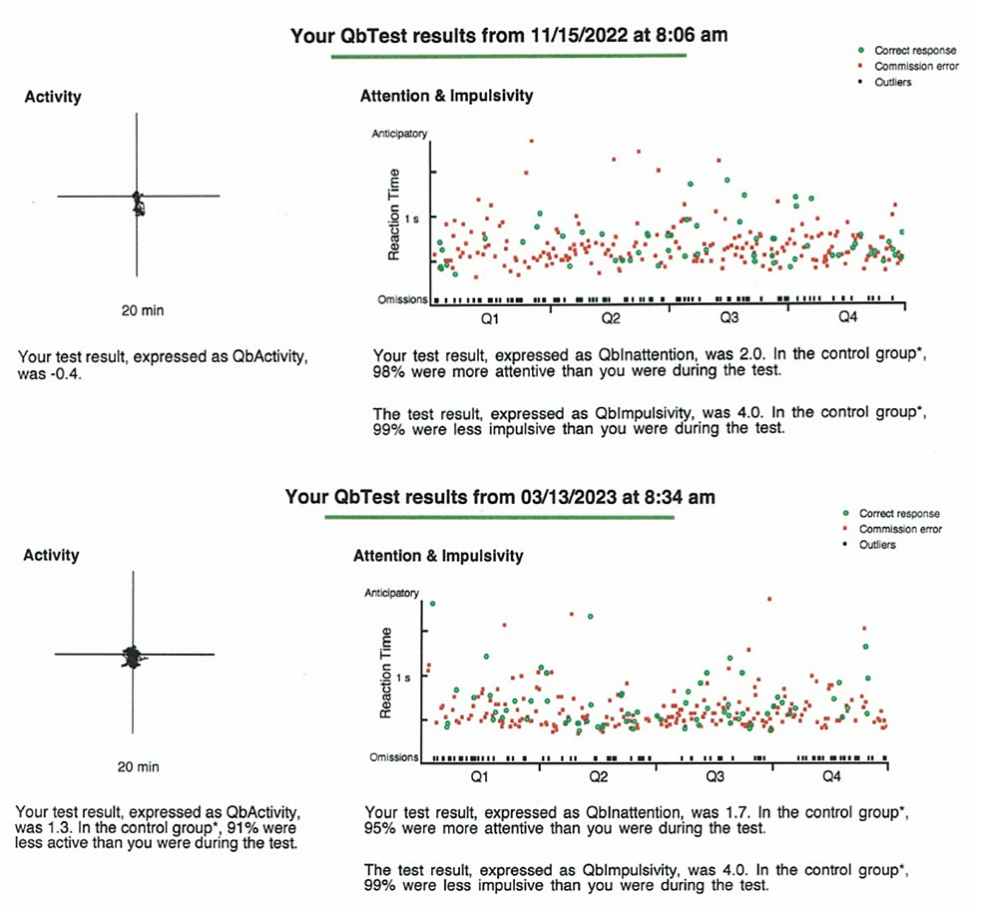
QbTest® maintained a dosage of lisdexamfetamine (30 mg).

In March 2023, the medical genetics report noted an additional physical exam finding of one abdominal café au lait spot. In addition, the results of a clinical exome sequence (XomeDx®Plus) analysis revealed a c.667 C>T p.(R223*) heterozygous pathogenic variant of the USP7 gene consistent with the diagnosis of HAFOUS. The patient continues to be managed symptomatically for ADHD and mild ID and continues to receive an IEP for school. The patient’s hyperactivity generally continues to be pharmacologically controlled well with corresponding results on QbTest® (Figure 2), but inattention and impulsivity continue to cause some difficulty.

There was an incident of an aggressive physical outburst on a family member in the summer of 2023; however, this incident occurred during a period in which the patient was not compliant with lisdexamfetamine (Vyvanse®) pharmacotherapy. Resources from the Foundation for USP7-Related Diseases were shared with the patient’s family, including information regarding an upcoming conference for families of individuals with HAFOUS. The patient continues to academically progress with an IEP; she is expected to graduate high school this year and expresses interest in attending vocational school afterward.

## Discussion

In this case, we present a female patient with HAFOUS who exhibited developmental delay, speech delay, and feeding difficulties during early childhood and currently exhibits mild ID and ADHD but has no current diagnosis of ASD. Anatomically, she exhibited an abdominal café au lait spot, small hands and feet, and facial dysmorphia, including deep-set eyes and low-set ears, without nasal anomaly or neurosensory impairment. In the literature review by Priolo et al. comparing previous clinical findings with their own patient, it was seen that 9/17 (53%) of patients exhibited ASD, 7/17 (41%) exhibited ADHD, 24/25 (96%) exhibited developmental delay/ID, 25/25 (100%) exhibited speech delay, 20/22 (91%) exhibited dysmorphic facies, 13/17 (76%) exhibited MRI anomalies, 16/22 (72%) exhibited hypotonia, 15/22 (68%) exhibited behavioral anomalies, 11/18 (61%) exhibited gastroesophageal reflux, 13/23 (56%) exhibited feeding difficulties, 16/25 (64%) exhibited vision impairment, additionally 25% of patients were reported to have small hands and feet [2]. It is interesting to note that Priolo et al. cited that with their own patient, performing behavioral studies was complicated by patient noncompliance with pharmacotherapy leading to temper tantrums, an event that also occurred with the patient described in our case report [2]. This notion, however, is also complicated due to the well-known phenomenon of amphetamine withdrawal, which in our case manifested as sporadic stimulant therapy noncompliance, which can cause agitation and aggression; therefore, it can be difficult to discern the extent to which aggression in a pharmacologically noncompliant group has a basis in withdrawal symptomatology rather than genetics [5]. There has also been documented literature of patients with HAFOUS having ovarian cancer, neurosensory hypoacusis, seizure disorder, polycystic kidney disease, and hepatosplenomegaly; however, the causality between these pathologies and USP7 mutations has not been clearly demonstrated but may provide further clinical utility in the diagnosis of HAFOUS [2,6,7].

An interesting aspect of this case was that developmental delay and ID in the context of facial dysmorphia raised suspicion for fragile X syndrome (FXS) by multiple physicians the patient had previously followed. This had prompted her first genetic test for SNP microarray with fragile X PCR five years prior to her current diagnosis, both of which had negative results, leading to an unclear diagnostic picture until this year when WES revealed the USP7 mutation, prompting her diagnosis of HAFOUS. Fragile X syndrome is a genetic condition diagnosed with FMR gene testing that less commonly occurs in females than males and carries a milder phenotypic expression in females, typically involving global developmental delays, anxiety disorders, ADHD, ASD, aggression, and socio-behavioral issues [8]. Common anatomic anomalies in FXS include facial narrowness with large ears and a prominent forehead. In our patient, however, the combination of deep-set eyes and low-set ears mimicked some of the phenotypic characteristics of FXS [9]; this may suggest that in patients with a phenotypic picture similar to FXS with contrary genetic testing, a differential diagnosis of HAFOUS should also be considered. Single nucleotide polymorphism microarrays are a commonly used technique in genetic analysis that focuses on detecting specific single nucleotide variations amongst a preset group of single nucleotide polymorphisms. They have proven effective in identifying known genetic variations. They are not used in the diagnosis of FXS but often accompany fragile X-PCR. Whole-exome sequencing is another common technique, but it involves sequencing the protein-coding exons of the genome. In contrast to SNP microarrays, it is more adept at identifying rare or novel genetic variations within the exome [10]. In a case report by Nickerson et al., it was discussed that the use of both SNP microarrays and WES together can be useful in the diagnosis of rare genetically heterogeneous disorders, such as their case involving autosomal recessive spastic ataxia of Charlevoix-Saguenay (ARSACS) [11]. Of note, however, HAFOUS is presently known to be a single-gene disorder, with documented cases being limited to the USP7 gene as deletions, nonsense mutations, frameshift indels, missense mutations, and splice site mutations [4]. In their paper, Fountain et al. elucidated a common phenotypic pattern amongst their subjects with HAFOUS, with all exhibiting speech delays and 18 of 20 exhibiting facial dysmorphism, additionally based on the MRI results of 11 of 15 individuals the authors included MRI anomalies as a common feature between cases in their discussion; however, it was noted that these signs and symptoms are nonspecific and highly variable between individuals [3,5]. Our patient in this case largely exhibited several common characteristics of HAFOUS described in the literature, namely facial dysmorphia, specifically referring to deep-set eyes, in addition to having small hands and feet, speech delay, childhood feeding difficulties, ID, ADHD, and aggression. Common features our patient did not exhibit were gastroesophageal reflux and visual impairment. An MRI was not performed on this patient as she did not have a history of craniocerebral trauma, malignancy, stroke, seizures, or neuromuscular deficits. Furthermore, our patient exhibited an abdominal café au lait spot, a skin macule that is often benign but can be associated with neurocutaneous genetic syndromes. It is currently unknown if HAFOUS is associated with this cutaneous finding, as it has not been documented in previous literature [12].

The patient in this case was never formally diagnosed with ASD, but she exhibited symptoms that may suggest this diagnosis. A similar observation in their patients was also noted by Fountain et al., possibly suggesting associated rates of ASD with HAFOUS are higher than currently recorded [4]. This case also offers an interesting insight not previously documented in prior literature on ADHD-associated HAFOUS; in this patient, we utilized pharmacogenomic reporting and analysis of QbTest® results in order to determine dose optimization of stimulant pharmacotherapy while simultaneously being able to quantifiably view inattention and impulsivity refractory to treatment. The aforementioned inattention and impulsivity may be attributed to a distinct neurogenetic etiology separate from inattentive ADHD, but further research is needed to fully delineate this association as well as the extent of impulsivity within subtypes of ADHD [13]. Additionally, the patient having had pharmacogenomic testing helped guide the decision to resume lisdexamfetamine (Vyvanse®) and in part explained previous unsuccessful attempts with dexmethylphenidate (Focalin®). Further research into the pharmacogenomics of patients with HAFOUS is needed in order to investigate a possible correlation between this condition and the genetic basis of drug responsiveness.

## Conclusions

Hao-Fountain syndrome is a rare neurodevelopmental disorder associated with mutations in the USP7 gene. Currently, diagnosis is based on the identification of a pathogenic variant of USP7 on WES. Our case report features a patient with a developmental history of delayed walking, speech delay, and feeding difficulties, as well as a neuropsychiatric history of ID and inattentive ADHD with anatomic anomalies consisting of an abdominal café au lait spot, low-set ears, deep-set eyes, and small hands and feet. While common phenotypic characteristics have been noted, clinical presentation is highly variable and nonspecific. Furthermore, HAFOUS appears to exhibit several behavioral and anatomical similarities to milder forms of FXS; therefore, it is important to have a multidisciplinary team consisting of practitioners from primary care, neuropsychology/psychiatry, and medical genetics involved in the diagnostics and management of HAFOUS. Currently, patients with HAFOUS are managed symptomatically, with further research needed with respect to the continuation of delineating common phenotypic characteristics as well as associations with other psychiatric and anatomic anomalies.

## References

[1] Hao YH, Fountain MD Jr, Fon Tacer K (2015). USP7 acts as a molecular rheostat to promote wash-dependent endosomal protein recycling and is mutated in a human neurodevelopmental disorder. Mol Cell.

[2] Priolo M, Mancini C, Pizzi S (2022). Complex presentation of Hao-fountain syndrome solved by exome sequencing highlighting co-occurring genomic variants. Genes (Basel).

[3] Zheng H, Mei S, Li F (2022). Expansion of the mutation spectrum and phenotype of USP7-related neurodevelopmental disorder. Front Mol Neurosci.

[4] Fountain MD, Oleson DS, Rech ME (2019). Pathogenic variants in USP7 cause a neurodevelopmental disorder with speech delays, altered behavior, and neurologic anomalies. Genet Med.

[5] Shoptaw SJ, Kao U, Heinzerling K, Ling W (2009). Treatment for amphetamine withdrawal. Cochrane Database Syst Rev.

[6] Zampieri N, Pulvirenti R, Pedrazzoli E, Camoglio FS (2022). Hao-Fountain syndrome and genital disorders: report of a new possible association. Ital J Pediatr.

[7] Scocchia A, Wigby KM, Masser-Frye D (2019). Clinical whole genome sequencing as a first-tier test at a resource-limited dysmorphology clinic in Mexico. NPJ Genom Med.

[8] Bartholomay KL, Lee CH, Bruno JL, Lightbody AA, Reiss AL (2019). Closing the gender gap in fragile X syndrome: review on females with FXS and preliminary research findings. Brain Sci.

[9] Ciaccio C, Fontana L, Milani D, Tabano S, Miozzo M, Esposito S (2017). Fragile X syndrome: a review of clinical and molecular diagnoses. Ital J Pediatr.

[10] Gabrielaite M, Torp MH, Rasmussen MS (2021). A comparison of tools for copy-number variation detection in germline whole exome and whole genome sequencing data. Cancers (Basel).

[11] Nickerson SL, Marquis-Nicholson R, Claxton K (2015). SNP analysis and whole exome sequencing: their application in the analysis of a consanguineous pedigree segregating ataxia. Microarrays (Basel).

[12] Lalor L, Davies OM, Basel D, Siegel DH (2020). Café au lait spots: when and how to pursue their genetic origins. Clin Dermatol.

[13] Baeyens D, Roeyers H, Walle JV (2006). Subtypes of attention-deficit/hyperactivity disorder (ADHD): distinct or related disorders across measurement levels?. Child Psychiatry Hum Dev.

